# Endoplasmic reticulum stress inhibits expression of genes involved in thyroid hormone synthesis and their key transcriptional regulators in FRTL-5 thyrocytes

**DOI:** 10.1371/journal.pone.0187561

**Published:** 2017-11-02

**Authors:** Gaiping Wen, Robert Ringseis, Klaus Eder

**Affiliations:** Institute of Animal Nutrition and Nutrition Physiology, Justus-Liebig-University Gießen, Gießen, Germany; University of Hong Kong, HONG KONG

## Abstract

Endoplasmic reticulum (ER) stress is characterized by the accumulation of misfolded proteins due to an impairment of ER quality control pathways leading to the activation of a defense system, called unfolded protein response (UPR). While thyrocytes are supposed to be highly susceptible to environmental conditions that cause ER stress due to the synthesis of large amounts of secretory proteins required for thyroid hormone synthesis, systematic investigations on the effect of ER stress on expression of key genes of thyroid hormone synthesis and their transcriptional regulators are lacking. Since the aim of the ER stress-induced UPR is to restore ER homeostasis and to facilitate cell survival through transient shutdown of ribosomal protein translation, we hypothesized that the expression of genes involved in thyroid hormone synthesis and their transcriptional regulators, all of which are not essential for cell survival, are down-regulated in thyrocytes during ER stress, while sterol regulatory element-binding proteins (SREBPs) are activated during ER stress in thyrocytes. Treatment of FRTL-5 thyrocytes with the ER stress inducer tunicamycin (TM) dose-dependently increased the mRNA and/or protein levels of known UPR target genes, stimulated phosphorylation of the ER stress sensor protein kinase RNA-like ER kinase (PERK) and of the PERK target protein eukaryotic initiation factor 2α (eIF2α) and caused splicing of the ER stress-sensitive transcription factor X-box binding protein (XBP-1) (P < 0.05). The mRNA levels and/or protein levels of genes involved in thyroid hormone synthesis, sodium/iodide symporter (NIS), thyroid peroxidase (TPO) and thyroglobulin (TG), their transcriptional regulators and thyrotropin (TSH) receptor and the uptake of Na^125^I were reduced at the highest concentration of TM tested (0.1 μg/mL; P < 0.05). Proteolytic activation of the SREBP-1c pathway was not observed in FRTL-5 cells treated with TM, whereas TM reduced proteolytic activation of the SREBP-2 pathway at 0.1 μg TM/mL (P < 0.05). In conclusion, the expression of key genes involved in thyroid hormone synthesis and their critical regulators and of the TSH receptor as well as the uptake of iodide is attenuated in thyrocytes during mild ER stress. Down-regulation of NIS, TPO and TG during ER stress is likely the consequence of impaired TSH/TSHR signaling in concert with reduced expression of critical transcriptional regulators of these genes.

## Introduction

One major function of the endoplasmic reticulum (ER) is to maintain the integrity of newly synthesized proteins by partitioning proteins between ER folding, trafficking and degradation pathways in a process called ER quality control [1]. Proteins that enter co-translationally the ER are folded into their functional conformations with the help of ER-localized chaperones and folding factors and then packaged into vesicles for trafficking to secretory environments. However, misfolded proteins are directed towards the ER-associated degradation (ERAD) pathway, where they are translocated to the cytosol and degraded by the ubiquitin proteasome system [2].

As a consequence of different environmental and genetic stressors, imbalances in ER quality control pathways can be induced leading to the accumulation of misfolded proteins, a condition termed ER stress [3]. In order to combat ER stress, eukaryotic cells from yeast to mammals have evolved a defense system, called unfolded protein response (UPR), which aims to re-establish ER quality control and to decrease ER accumulation of misfolded proteins [4,5]. The UPR is a tripartite signaling pathway activated by three transmembrane ER proteins sensing the accumulation of misfolded proteins: ATF6 (activating transcription factor 6), IRE1 (inositol-requiring protein 1a) and PERK (protein kinase RNA-like ER kinase). One important effect of the UPR, which is mediated by PERK-dependent phosphorylation/inactivation of eukaryotic initiation factor 2α (eIF2α), is transient attenuation of new protein synthesis leading to a decrease of newly synthesized proteins entering the ER, thereby reducing the load of ER folding and degradation pathways and enabling the ER to better cope with misfolded proteins accumulating during ER stress [5,6]. ER protein folding load is also reduced through an IRE1-dependent mRNA degradation mechanism abbreviated as RIDD (regulated IRE1-dependent decay of mRNA) [7], thereby, leading to a decrease of proteins entering co-translationally the ER. A further important effect mediated by IRE1 is unconventional mRNA splicing of X-box binding protein (XBP)-1, which leads to the production of the active transcription factor sXBP-1 inducing genes involved in chaperoning, disulfide isomerization, N-glycosylation and vesicular trafficking [8]. ER quality control capacity is also improved as a consequence of the UPR due to activation of ATF6, a transcription factor inducing genes involved in protein folding and degradation [9].

Thyrocytes are highly specialized polarized epithelial cells that synthesize large amounts of secretory proteins required for thyroid hormone synthesis and are therefore particularly susceptible to environmental conditions that cause ER stress, as shown recently [10,11]. Indeed, evidence exists that ER stress/UPR plays a pathophysiological role in different thyroid disorders including congenital hypothyroid goiter [11–13], cystinosis-induced hypothyroidism [14] and amiodarone-induced destructive thyroiditis [15]. In line with this, it has been reported that ER stress impairs glycosylation of thyroglobulin (TG), a major secretory protein of the thyrocyte, and ER retention of TG [10]. TG is a large glycoprotein secreted from the thyrocyte into the follicular lumen [16], where selected tyrosyl residues of TG become iodinated leading to the thyroid hormone precursors mono- and diiodotyrosine [17]. While the effect of ER stress on TG expression in thyrocytes has been extensively studied [10], systematic investigations on the effect of ER stress on expression of key genes of thyroid hormone synthesis and their transcriptional regulators are lacking in the literature. Besides TG, sodium/iodide symporter (NIS) and thyroid peroxidase (TPO) play a central role for thyroid hormone synthesis as evidenced from the fact that genetic abnormalities in any of these key proteins leads to severe hypothyroidism [18–20]. Regulation of thyrocyte gene expression of TG, NIS and TPO is under the control of thyrotropin (TSH), the main hormonal regulator of the thyroid, and involves the transcription factors thyroid transcription factor (TTF)-1, forkhead box E1 (FOXE1, also named TTF-2) and paired box-8 (PAX-8) [21–25]. Since the aim of the ER stress-induced UPR is to restore ER homeostasis and to facilitate cell survival through transient shutdown of ribosomal translation of mRNAs and enhancing the capacity of ER protein folding and degradation pathways, we hypothesized that the expression of genes involved in thyroid hormone synthesis and their transcriptional regulators, all of which are not essential for cell survival, are down-regulated in thyrocytes during ER stress.

We have recently reported that sterol regulatory element-binding proteins (SREBP)-1c and SREBP-2, which have been initially identified as master regulators of genes involved in lipid synthesis and uptake such as fatty acid synthase (FASN), glycerol-3-phosphate acyltransferase, mitochondrial (GPAM), 3-hydroxy-3-methylglutaryl-CoA reductase (HMGCR) and LDL receptor (LDLR) [26], also act as transcriptional regulators of NIS, TPO and TG and are under the control of TSH in thyrocytes [27–29]. Despite the role of SREBPs as transcriptional regulators of NIS, TPO and TG, we hypothesized that SREBPs are activated in thyrocytes during ER stress. This is based on observations from different groups that SREBP-1c and SREBP-2 are activated in different cell types under ER stress conditions [30–35]. Activation of SREBPs during ER stress is mediated by phosphorylation of the ER stress sensor PERK and is considered as a means to synthesize membrane lipids, like fatty acids and cholesterol, which are required for the expansion of the ER during ER stress [36]. To test our hypotheses, we used Fisher rat thyroid cell line-5 (FRTL-5) cells, a frequently used thyrocyte model dependent on thyrotropin (TSH) [37], which were treated with tunicamycin (TM). TM is a chemical compound inducing ER stress through inhibiting protein glycosylation [38].

## Material and methods

### Cell culture

FRTL-5 cells were obtained from Cell lines service (Eppelheim, Germany). Cells were cultured in Ham´s F12 medium supplemented with 5% newborn calf serum, 1% antibiotic-antimycotic-mixture and a six-hormone mixture as described recently [29]. Medium was changed every two days. After reaching a confluence of 70–80%, the cells were either sub-cultivated or used for experiments.

### Cell viability assay

The 3-(4,5-dimethylthiazol-2-yl)-2,5-diphenyltetrazolium bromide (MTT) assay was used to assess cell viability and performed according to the manufacturer´s directions (Sigma). In brief, FRTL-5 cells were seeded in 96-well plates at a density of 5 x 10^4^ cells per well. After reaching confluence, cells were treated with different concentrations of TM (Sigma-Aldrich, Taufkirchen, Germany) as indicated for 24 h. Control cells were treated with the same concentration of vehicle (DMSO). Cells treated with medium only were used to evaluate a possible effect of DMSO. Following treatment, medium was aspirated and cells were incubated with MTT solution (1 mg/mL PBS) for 4 h. Finally, formazan crystals were dissolved and absorbance was measured as described recently [39]. Cell viabilities are expressed as percentage of control cells (viability of control cells was set to 100%). Mean absorbance value of vehicle control was set to 100%, and the means and SD of absorbance values of TM-treated cells was presented relative to that of vehicle control. Three independent experiments were run.

### RNA isolation and quantitative real-time RT-PCR

For qPCR experiments, FRTL-5 cells were seeded in 24-well plates at a density of 1 x 10^5^ cells per well and treated, after reaching confluence, with the TM concentrations indicated for 24 h. Control cells indicated as “0” were treated with the same concentration of vehicle (DMSO). Following treatment, the medium was aspirated, the cell layer washed with PBS, and plates immediately stored at -80°C until RNA extraction. Total RNA was extracted from cells using Trizol^™^ reagent (Invitrogen, Karlsruhe, Germany) according to the manufacturer´s protocol. The cDNA was synthesized as described recently in detail [40]. qPCR analysis on a Rotor-Gene Q system (Qiagen, Hilden, Germany) and calculation of mRNA levels was performed as described recently in detail [39]. Gene-specific primer pairs were designed using Primer3 [41] and BLAST [42] and synthesized by Eurofins MWG Operon (Ebersberg, Germany) (Table 1). For normalization of relative expression values of target genes the method of Vandesompele et al. [43] was used. In the present study four potential reference genes were tested (TOP1, RPL13, ACTB, WYHAZ), from which three (TOP, RPL13, ACTB; M-values between 0.399 and 0.430; V-value V3/V4 = 0.124) were used to calculate the normalization factor as the geometric mean of their expression data. Within each independent experiment, all technical replicates were analyzed and a single mean was calculated for each treatment. The mean of vehicle control (“0”) was set to 1.0 and the mean and SD of the TM-treated cells were scaled proportionally in order to show the changes in mRNA levels as fold of vehicle control. Three independent experiments were run.

**Table 1:** Sequences of gene-specific primers used for quantitative real-time PCR analysis.

Gene name	Primer sequence (forward, reverse)	Product size (bp)	NCBI GenBank
*Reference genes*			
ACTB	GACCTCTATGCCAACACAGT CACCAATCCACACAGAGTAC	154	NM_031144
RPL13	CTTAAATTGGCCACGCAGCT CTTCTCAACGTCTTGCTCTG	198	XR_086310
TOP1	GAAGAACGCTATCCAGAAGG GCTTTGGGACTCAGCTTCAT	137	NM_022615
YWHAZ	GACGGAAGGTGCTGAGAAA GCAGCAACCTCAGCCAAGT	198	NM_013011
*Target genes*			
ATF4	AACACAGCCCTTCCACCTCC TGCTCAGCCCTCTTCTTCTGG	205	NM_024403
BAX	AGGCGAATTGGCGATGAACT GAAGCCTCAGCCCATCTTCTTC	396	NM_017059
CHOP	ACAAGCACCTCCCAAAGCCC TGCTCCTTCTCCTTCATGCGC	155	NM_001109986
BiP	TCAGCCCACCGTAACAATCAAGG TCCTCAGCAAACTTCTCGGCG	282	NM_013083
FASN	AGGTGCTAGAGGCCCTGCTA GTGCACAGACACCTTCCCAT	281	X62888
GPAM	CAGCGTGATTGCTACCTGAA CTCTCCGTCCTGGTGAGAAG	194	U36771
HMGCR	CGAGCAAGTGATTACCCTGA GTCTTGGTTCACTCCTGGAT	223	NM_013134
LDLR	ACAGTGTCCTCCCAAGTCCAA GCAAATGTGGATCTCGTCCTC	221	NM_175762
NIS	GCTGTGGCATTGTCATGTTC TGAGGTCTTCCACAGTCACA	219	NM_052983
PAX-8	CCTTACTCAACAGTACCCTGG AGCTAGAACTGGAGAGCTCTG	162	NM_031141
XBP-1/sXBP-1	GACACGCTTGGGGATGAATGC AGAGGCAACAGCGTCAGAATCC	192/166	NM_001004210/NM_001271731
SREBP-1c	GGAGCCATGGATTGCACATT AGGAAGGCTTCCAGAGAGGA	191	NM_001276708
SREBP-2	CTGACCACAATGCCGGTAAT CTTGTGCATCTTGGCATCTG	204	NM_001033694
TG	GTTCCTACGTGTACTAGTGAG CATACTGGAGTTGGAGAGCAG	196	NM_030988
TPO	CAGGTGTTGAGAAGCAGTTG CTTTGAAAGCTGTAGCCAGG	255	NM_019353
TSHR	CCAGAAGCTTGACTTACATAG CATGTAAGGGTTGTCTGTGAT	161	NM_012888
TTF-1	GCATGAATATGAGCGGCATGG ACTTCTGCTGCTTGAAGCGTC	153	NM_013093
TTF-2	GAAGTGGCAGAACAGCATCC	139	NM_138909

### Immunoblotting

For immunoblotting experiments, FRTL-5 cells were seeded in 6-well plates at a density of 2 x 10^5^ cells per well and treated, after reaching confluence, with the TM concentrations indicated for 24 h. Control cells indicated as “0” were treated with the same concentration of vehicle (DMSO). Following treatment, the medium was aspirated, the cell layer washed with PBS and either cell lysates or subcellular fractions (nuclear fraction, cytosolic fraction) were prepared. Within each independent experiment, two technical replicates per treatment were pooled during preparation of cell lysates and subcellular fractions. For detection of BiP, CHOP, PERK, eIF2α, TG, NIS, TTF-1, TTF-2 and PAX-8, cell lysates were prepared with radioimmunoprecipitation assay (RIPA) lysis buffer [50 m*M* Tris (pH 7.5), 150 m*M* NaCl, 1 m*M* EDTA, 1% Triton X-100, 0.1% SDS, and 1% sodium deoxycholate] containing protease inhibitors (Sigma-Aldrich Chemie GmbH, Germany). For detection of p-PERK, p-eIF2α and p-TG, cell lysates were prepared with RIPA lysis buffer containing the phosphatase inhibitors sodium fluoride (5 mM) and sodium-orthovanadat (1 mM) (both from Sigma-Aldrich) as well as protease inhibitors (Sigma-Aldrich). For detection of nSREBP-1/2 and pSREBP-1/2, nuclear and cytosolic extracts, respectively, were prepared with the Nuclear Extract Kit from Active Motif (La Hulpe, Belgium) according to the manufacturer’s protocol. Determination of protein concentrations of cell lysates and subcellular fractions, separation of proteins by SDS-PAGE and electrotransfer to nitrocellulose membranes and blocking of membranes were carried out as described recently (Chiappisi et al. 2017). Afterwards, membranes were incubated with primary antibodies against SREBP-1 (1:300, Santa Cruz Biotechnology, Heidelberg, Germany), SREBP-2 (1:300, Santa Cruz Biotechnology), NIS (1:2,000, kindly provided from Prof. N. Carrasco, New Haven, USA), TG (1:10000, Abcam, Cambridge, UK), BiP (1:5,000, Thermo Fisher Scientific, Langenselbold, Germany), total and p-eIF2α (both 1:1,000, Cell Signaling Technology, Danvers, MA, USA), total and p-PERK (both 1:1,000, Signaling Technology), CHOP (1:2,000, Thermo Fisher Scientific), TTF-1 (1:500, Santa Cruz Biotechnology), TTF-2 (1:500, Santa Cruz Biotechnology), PAX-8 (1:500, Santa Cruz Biotechnology), and either β-actin (1:40.000, Abcam) or vinculin (1:10,000, Thermo Fisher Scientific) as a reference protein for normalization at 4°C overnight. The membranes were washed and then incubated with a horseradish peroxidase-conjugated secondary anti-rabbit-IgG (1:10,000, Sigma-Aldrich) or anti-mouse-IgG antibody (1:10,000, Santa Cruz Biotechnology) at RT for 1.5 h. Chemiluminescence detection and quantification of signal intensities of specific bands was carried out as described [39]. For calculation of protein levels, the band intensity of the proteins of interest was normalized by that of β-actin. Within one independent experiment, all technical replicates per treatment were analyzed and a single mean was calculated for each treatment. The mean of vehicle control (concentration “0”) was set to 1.0 and the mean and SD of the other concentrations were scaled proportionally in order to show the changes in protein expression as fold of vehicle control. Three independent experiments were run.

### Iodide uptake

For iodide uptake, FRTL-5 cells were seeded in 24-well plates at a density of 1 x 10^5^ cells per well and treated, after reaching confluence, with the TM concentrations indicated for 24 h. The uptake assay was performed as described recently [44]. In brief, the medium was removed, cells were washed with warm (37°C) Hank's buffered salt solution (HBSS) and then incubated with HBSS containing Na^125^I (0.3 μCi/mL) at 37°C for 1 h. To distinguish between total iodide uptake and NIS-specific uptake, the incubation was performed both in the absence (total iodide uptake) and the presence (NIS-specific uptake) of 1 mM KClO4, a specific inhibitor of iodide uptake via NIS. The incubation was terminated by aspiration of the medium. After rinsing two times with ice-cold HBSS, cells were lysed with 0.5 mL 0.5 M NaOH with shaking at RT for 30 min. The cell lysate from each well was transferred in a counter vial for scintillation counting (Perkin Elmer Liquid Scintillation Analyzer Tri-Carb 2900TR, Rodgau, Germany). Na^125^I uptake is presented as counts per minute and well. Three independent experiments were run.

### Statistical analysis

All data represent means and SD. In all figures showing numerical data, with the exception of data from Na^125^I uptake, the means and SD were calculated from the means for the same treatments of three independent experiments. Data were analyzed by Student´s t-test comparing cells of each TM concentration with the vehicle control cells (“0”) using the Minitab Statistical Software Rel. 13.0 (Minitab, State College, PA, USA). Differences of P < 0.05 were considered as significant.

## Results and discussion

### ER stress induced by TM impairs cell viability and activates the unfolded protein response in FRTL-5 cells

**Fig 1:**
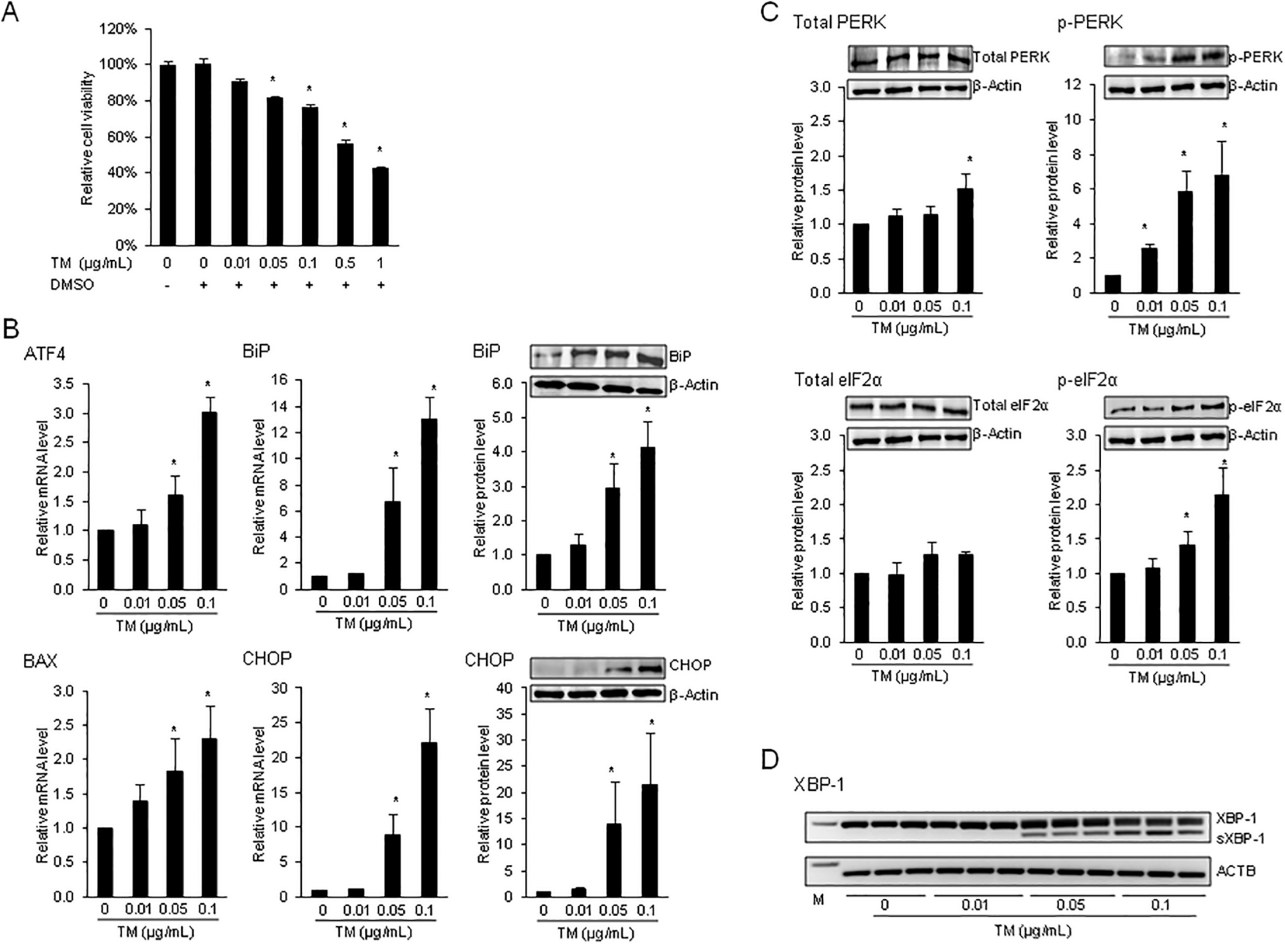
ER stress inducer tunicamycin impairs cell viability and activates the unfolded protein response in FRTL-5 cells. Effect of 24-h treatment of FRTL-5 thyrocytes with different concentrations of tunicamycin (TM) on: (A) cell viability, (B) mRNA and/or protein levels of the unfolded protein response (UPR) target genes activating transcription factor 4 (ATF4) 4, BCL2 associated X, apoptosis regulator (BAX), glucose-regulated protein, 78kDa (GRP78/BiP) and C/EBP homologous protein (CHOP), (C) protein levels of protein kinase RNA-like ER kinase (PERK) and p-PERK, eukaryotic initiation factor 2α (eIF2α) and p-eIF2α, and (D) splicing of X-box binding protein (XBP)-1. (A-D) Cells treated with DMSO used as vehicle served as control. (A) Bars represent cell viability relative to control (0 μg/mL TM), which was set to 100%, and are means ± SD from 3 independent experiments. (B, C) Bars represent relative mRNA or protein levels expressed as fold of control (0 μg/mL TM), which was set to 1.0, and are means ± SD from 3 independent experiments. One representative immunoblot for BAX, BiP, CHOP, PERK, p-PERK, eIF2α and p-eIF2α and β-Actin from three independent experiments is shown. (D) Representative image of XBP-1 activation by unconventional splicing in FRTL-5 cells demonstrating the unspliced (192 bp) and the spliced (s; 166 bp) XBP-1 mRNA as detected by conventional PCR followed by agarose gel electrophoresis. Actin beta (ACTB) was detected by conventional PCR and served as a loading control for agarose gel electrophoresis. *Different from control (P < 0.05).

Although the aim of the ER stress-induced UPR is to restore ER homeostasis and to facilitate cell survival through transient shutdown of protein synthesis and enhancing the capacity of ER quality control pathways, the UPR can also activate the apoptotic program of cell death if ER stress persists chronically at high level and ER homeostasis cannot be restored [45,46]. Since apoptosis *per se* interfere with other cellular functions, it is critical to investigate the cellular response to ER stress inducers at a dose at which apoptosis is avoided, yet ER stress signaling pathways are activated. Thus, at the beginning of the study the viability of FRTL-5 cells was assessed in response to 24 h incubation with increasing concentrations of TM (from 0.01 to 1 μg/mL). Incubation with TM reduced FRTL-5 cell viability by 10% at the lowest concentration (not significant) and by 55% at the highest concentration tested (P < 0.05; Fig 1A). At concentrations >0.01 and <0.5 μg/mL cell viability was impaired by approximately 20% (P < 0.05). Based on this, the response of FRTL-5 cells to TM in the subsequent experiments was investigated at concentrations from 0.01 to 0.1 μg TM/mL.

Induction of ER stress by TM in FRTL-5 cells was evaluated by determining the mRNA and/or protein levels of several ER stress/UPR target genes, the phoshorylation of ER stress sensor PERK and its downstream phosphorylation target eIF2α and the splicing of XBP-1, all of which are indications of ER stress [47]. As shown in Fig 1B, the mRNA and/or protein levels of the UPR target genes activating transcription factor 4 (ATF4), BCL2 associated X, apoptosis regulator (BAX), glucose-regulated protein, 78kDa (GRP78/BiP) and C/EBP homologous protein (CHOP) were induced by 24 h treatment with TM at concentrations of 0.05 μg/mL and above, whereas no response to TM was observed at a TM concentration of 0.01 μg/mL. Induction of all UPR target genes tested was greater at 0.1 μg TM/mL than at 0.05 μg/mL. In line with the effect of TM on UPR target gene induction, the relative protein level of p-PERK was increased by TM at concentrations of 0.05 μg/mL and above by 6–7 fold (P < 0.05), whereas the protein level of total PERK was largely unchanged (Fig 1C). Phosphorylation of PERK and thus initiation of PERK signaling is a key event of ER stress induction. It is mediated by dissociation of BiP from the PERK sensing domain localized to the ER lumen and thereby leads to the activation/phosphorylation of PERK through a mechanism involving autophosphorylation of the cytosolic PERK kinase domain. Subsequently, the active, p-PERK kinase selectively phosphorylates the serine 51 residue of eIF2α [48] leading to inactivation of eIF2α and thus inhibition of ribosomal translation initiation and transient attenuation of new protein synthesis [49]. Thus, phosphorylation of eIF2α is a further sensitive indicator of ER stress and activation of the UPR. In agreement with PERK phosphorylation by TM, the relative protein level of p-eIF2α was elevated at 0.05 and 0.1 μg TM/mL (P < 0.05; Fig 1C), but not at 0.01 μg/mL, whereas the protein level of total eIF2α was not influenced by TM. Finally, ER stress induction by TM in FRTL-5 cells was evidenced from the occurrence of unconventional splicing of XBP-1, another key event of ER stress signaling caused by the ER stress transducer IRE1. Subsequent translation of sXBP-1 mRNA into the active transcription factor sXBP-1 causes activation of promoters of genes encoding ER chaperones and ERAD components [8]. ER chaperones and ERAD components help to restore ER homeostasis through facilitating protein folding and attenuating accumulation of misfolded proteins [5,6]. As shown in Fig 1D, a 166-bp PCR product representing the sXBP-1 mRNA could be observed in FRTL-5 cells treated with 0.05 and 0.1 μg TM/mL. No sXBP-1 mRNA was observed in FRTL-5 cells treated with 0.01 μg TM/mL and in control cells. These results clearly showed that TM induces ER stress in FRTL-5 cells in a dose-dependent manner.

### ER stress attenuates the expression of genes involved in thyroid hormone synthesis and reduces iodide uptake in FRTL-5 cells

**Fig 2:**
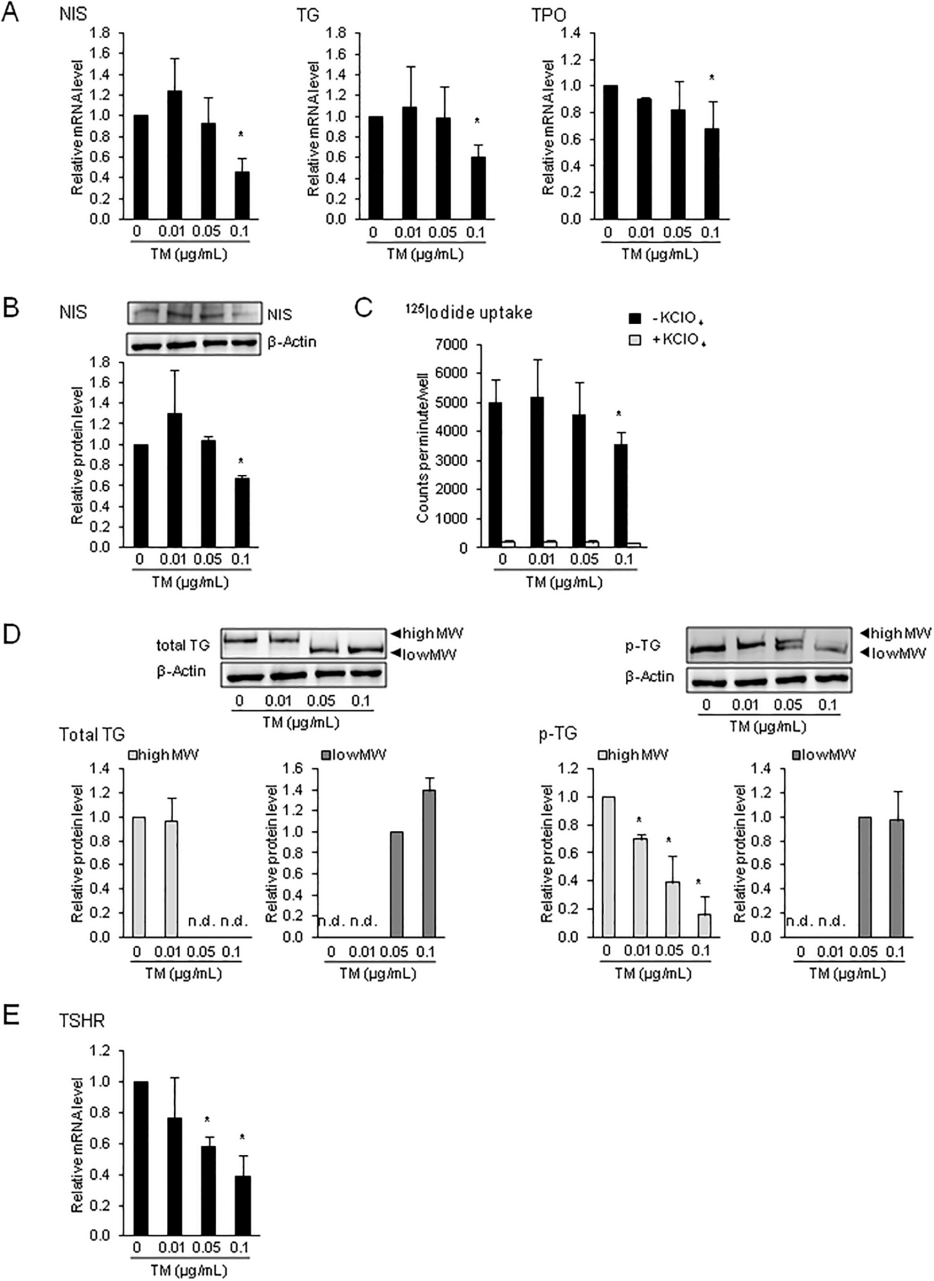
ER stress attenuates the expression of genes involved in thyroid hormone synthesis and reduces iodide uptake in FRTL-5 cells. Effect of 24-h treatment of FRTL-5 thyrocytes with different concentrations of tunicamycin (TM) on: (A) relative mRNA levels of sodium/iodide symporter (NIS), thyroglobulin (TG) and thyroid peroxidase (TPO), (B) protein level NIS, (C) Na^125^I uptake in the presence and absence of the NIS-specific inhibitor KClO_4_, (D) protein levels of total and p-TG, and (E) relative mRNA level of TSH receptor (TSHR). (A-E) Cells treated with DMSO used as vehicle served as control. (A, B, D, E) Bars represent relative mRNA or protein levels expressed as fold of control (0 μg/mL TM), which was set to 1.0, and are means ± SD from 3 independent experiments. (C) Bars represent counts per minute/well and are means ± SD from 3 independent experiments. (D) One representative immunoblot for total TG, p-TG and β-Actin from three independent experiments is shown. *Different from control (P < 0.05).

The effect of ER stress on expression of genes involved in thyroid hormone synthesis was investigated in FRTL-5 cells treated for 24 h with the same TM concentrations as above. As shown in Fig 2A, the mRNA levels of NIS, TG and TPO were reduced by about 30 to 50% by TM at a concentration of 0.1 μg/mL (P < 0.05). This result is in agreement with earlier observations that different ER stress inducers decrease the expression of genes involved in thyroid hormone synthesis, like TG [10] and NIS [50]. At concentrations of TM ≤ 0.05 μg TM/mL, which were found to cause ER stress, no effect on expression of these genes was observed. This indicates that the expression of genes involved in thyroid hormone synthesis in thyrocytes is attenuated only during stronger ER stress but not during mild ER stress.

The inhibitory effect of TM on NIS mRNA level was also translated to the protein level as demonstrated by the finding that the protein level of NIS was also reduced by the high concentration of TM by approximately 30% as compared to vehicle control cells (P < 0.05; Fig 2B). To study whether the decreased protein level of NIS reduces iodide uptake into FRTL-5 cells during ER stress, the effect of TM was also investigated on Na^125^I uptake into FRTL-5 cells, both in the presence and absence of KClO4 (1 mM), a specific inhibitor of iodide uptake via NIS. In line with the decreased NIS expression by 0.1 μg TM/mL, total iodide uptake (absence of KClO_4_) into FRTL-5 cells was reduced by 24 h treatment with TM at 0.1 μg/mL by about 30% (P < 0.05; Fig 2C), whereas lower concentrations of TM had no effect (Fig 2C). In the presence of KClO4 in the culture medium, TM had no effect on Na^125^I uptake indicating that TM inhibits NIS-specific iodide uptake.

While the inhibitory effect of TM on TPO mRNA level could not be verified at the protein level due to the lack of an appropriate TPO-specific antibody, the effect of TM was also studied on TG protein level. Immunoblotting with a TG-specific antibody revealed the detection of a protein with a smaller size in FRTL-5 cells treated with 0.05 and 0.1 μg TM/mL than in control cells and cells treated with 0.01 μg TM/mL (Fig 2D), in which a larger TG was detected. This observation has been made earlier in FRTL-5 cells treated with TM [10,11] and is explained by the fact that TM inhibits protein glycosylation [38]. Thus, the newly synthesized TG, which accounts for greater than 50% of newly synthesized cargo proteins of thyrocytes [51] and becomes glycosylated in the ER of the thyrocyte through addition of *N*-linked oligosaccharide chains [52], was obviously not glycolysated in FTRL-5 cells treated with the high concentrations of TM leading to a smaller (unglycosylated) TG protein. Besides glycosylation, TG undergoes posttranslational modification by phosphorylation of carbohydrate chains (50%) and serine (30%) and tyrosine residues (20%) [53]. Phosphorylation has been suggested to take place in the Golgi complex and is thought to be important for directing TG through the Golgi complex to the endosomes and, finally, to the lysosomes [54]. To study the effect of ER stress on phosphorylation of TG in FRTL-5 cells, proteins were isolated also from TM-treated FRTL-5 cells using a buffer containing phosphatase-inhibitors. Fig 2D shows that in FRTL-5 cells treated with TM concentrations of ≥ 0.05 μg/mL the amount of p-TG with the high molecular size decreased compared to control cells and cells treated with 0.01 μg TM/mL. A phosphorylated form of TG with a lower molecular size was found only in cells treated with the high TM concentrations, but the bands for the low molecular p-TG were markedly less strong than those of high molecular size p-TG. These findings suggest that TM treatment of FRTL-5 cells not only inhibits TG glycosylation, but also TG phosphorylation, despite the fact that TM obviously decreased TG availability for phosphorylation because of inhibition of TG glycosylation which precedes phosphorylation.

To further study whether ER stress also affects the expression of the TSH receptor (TSHR), which mediates the stimulatory effect of TSH on the expression of NIS, TG and TPO at the transcriptional and/or posttranslational level [55,56], we determined the mRNA level of TSHR. As shown in Fig 2E, treatment with TM at concentrations > 0.01 μg/mL reduced the expression of TSHR in FRTL-5 (P < 0.05). Although we did not analyze key signaling proteins downstream of TSHR such as protein kinase A, our observation suggests that TSH signaling in the thyrocyte is likely impaired during ER stress and the impaired TSH signaling may contribute to the decreased expression of NIS, TG and TPO.

### ER stress has divergent effects on transcriptional regulators of genes involved in thyroid hormone synthesis in FRTL-5 cells

**Fig 3:**
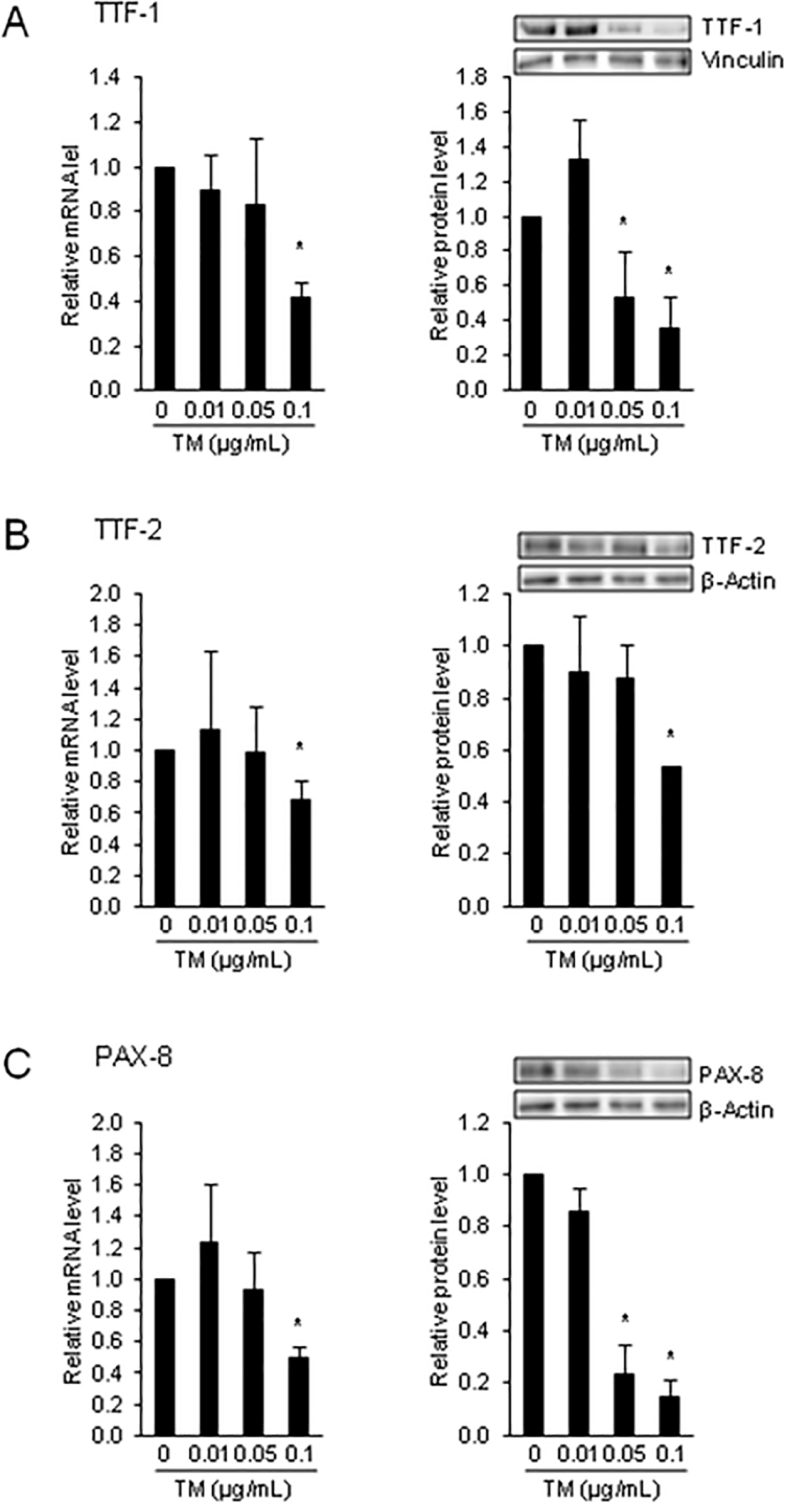
ER stress reduces the expression of critical regulators of genes involved in thyroid hormone synthesis in FRTL-5 cells. Effect of 24-h treatment of FRTL-5 thyrocytes with different concentrations of tunicamycin (TM) on relative mRNA and protein levels of: (A) (TTF)-1, (B) forkhead box E1 (FOXE1, also named TTF-2), and (C) paired box-8 (PAX-8). (A-C) Cells treated with DMSO used as vehicle served as control. Bars represent relative mRNA or protein levels expressed as fold of control (0 μg/mL TM), which was set to 1.0, and are means ± SD from 3 independent experiments. (A-C) One representative immunoblot for TTF-1, TTF-2, PAX-8, β-Actin and vinculin is shown. *Different from control (P < 0.05).

Transcriptional regulation of NIS, TPO and TG by TSH was found to involve binding sites for TTF-1, TTF-2 and PAX-8 [21–25]. To study whether the inhibitory effect of ER stress of gene expression of NIS, TPO and TG is accompanied by a decreased expression of these transcription factors, the mRNA and protein levels of TTF-1, TTF-2 and PAX-8 were determined in FRTL-5 cells treated with TM. As shown in Fig 3A–3C, the mRNA and protein levels of all three TSH-dependent transcription factors were reduced in cells treated with 0.1 μg TM/mL (the protein levels of TTF-1 and PAX-8 even at 0.05 μg TM/mL) (P < 0.05), but not in cells treated with lower TM concentrations and in control cells. These findings indicated that the reduced expression of genes involved in thyroid hormone synthesis in response to ER stress induction in FRTL-5 cells involves down-regulation of critical regulators of these genes. Whether this effect is due to the impaired TSHR expression/TSH signaling or due to a direct effect of ER stress on the expression of TTF-1, TTF-2 and PAX-8 cannot be answered from the present data and requires further investigations.

Despite the well known role of TTF-1, TTF-2 and PAX-8 in regulating genes involved in thyroid hormone synthesis, we have recently identified SREBP-1c and SREBP-2 as novel transcriptional regulators of NIS, TPO and TG [27–29]. Using reporter gene, DNA binding and chromatin immunoprecipitation assays, we could identify specific SREBP binding sites, called sterol response elements (SRE), within different regulatory regions of NIS, TPO and TG [27–29]. While SREBPs were initially discovered as master transcription factors of cholesterol, fatty acid, triacylglycerol and phospholipid synthesis [26], the data from our group indicated that SREBPs have functions beyond lipid synthesis, such as hormone synthesis, at least in the thyroid. Interestingly, we have also found recently that expression and activation of SREBP-1c and SREBP-2 is stimulated by TSH in FRTL-5 thyrocytes [27], an effect that mechanistically explains earlier observations that TSH stimulates the synthesis of cholesterol and fatty synthesis in thyrocytes [57–59]. Since these lipids are important membrane constituents required for thyrocyte growth and proliferation both of which are stimulated by TSH, we have proposed recently that TSH-dependent stimulation of SREBPs aims to coordinate lipid and thyroid hormone synthesis in growing and proliferating thyrocytes [27].

**Fig 4:**
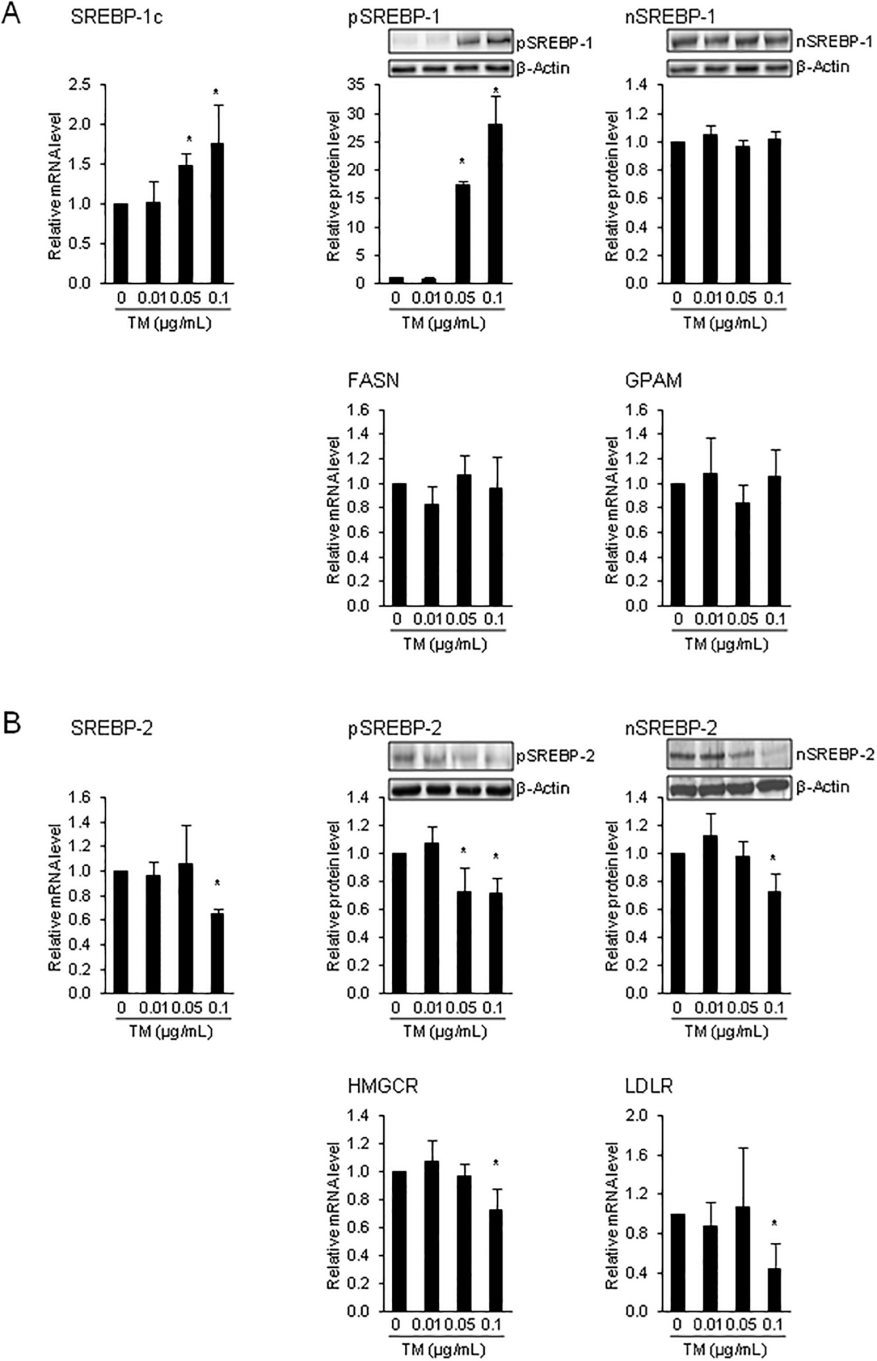
ER stress has divergent effects on SREBP-1c and SREBP-2 pathway in FRTL-5 cells. Effect of 24-h treatment of FRTL-5 thyrocytes with different concentrations of tunicamycin (TM) on: (A) relative mRNA levels of sterol regulatory element-binding protein-1c (SREBP-1c) and its target genes fatty acid synthase (FASN) and glycerol-3-phosphate acyltransferase, mitochondrial (GPAM), and on protein levels of precursor (p) and nuclear (n) SREBP-1, and (B) relative mRNA levels of sterol regulatory element-binding protein-2 (SREBP-2) and its target genes 3-hydroxy-3-methylglutaryl-CoA reductase (HMGCR) and LDL receptor (LDLR) and on protein levels of precursor (p) and nuclear (n) SREBP-2. (A, B) Cells treated with DMSO used as vehicle served as control. Bars represent relative mRNA or protein levels expressed as fold of control (0 μg/mL TM), which was set to 1.0, and are means ± SD from 3 independent experiments. (A, B) One representative immunoblot for n/pSREBP-1, n/pSREBP-2 and β-Actin is shown. *Different from control (P < 0.05).

Several studies using cells from non-thyroidal tissues demonstrated that ER stress causes proteolytic activation of SREBP-1c and thereby promotes *de novo* lipogenesis [33,34]. The physiological meaning of the ER stress-induced activation of SREBP-1c pathway is that the expansion of the ER during the UPR requires an additional provision of fatty acids in order to synthesize the necessary phospholipids. In contrast to these observations from non-thyroidal tissues/cells, we found that TM at concentrations > 0.01 μg/mL increased the mRNA level of SREBP-1c and the protein level of the inactive pSREBP-1, but did not increase the protein level of the transcriptionally active nSREBP-1 (Fig 4A). In line with the latter, the mRNA levels of two known SREBP-1c target genes, FASN and GPAT, were not increased by TM. These results indicated that proteolytic activation of SREBP-1c is not stimulated during ER stress in FRTL-5 thyrocytes, despite strong transcriptional activation of the SREBP-1c gene under ER stress. We cannot explain the lack of proteolytic activation of SREBP-1 in FRTL-5 thyrocytes under ER stress conditions, but it may be speculated that this is due to the impairment of TSH/TSHR signaling because we have recently shown that TSH stimulates proteolytic processing of pSREBP-1 in FRTL-5 cells [27].

Our hypothesis with regard to the effect of ER stress on SREBP activation could also not be confirmed in the case of SREBP-2, which is mainly responsible for regulating genes involved in cholesterol synthesis and uptake [26]. Obviously, expression and proteolytic activation of SREBP-2 is reduced during ER stress in FRTL-5 cells, as evident from decreases of the mRNA level of SREBP-2 and protein levels of pSREBP-2 and nSREBP-2 and the reduced mRNA levels of the SREBP-2 target genes HMGCR and LDLR following treatment with the highest concentration of TM tested (P < 0.05; Fig 4B). Since SREBP-2 was recently described as a transcriptional regulator of NIS, TG and TPO [27–29], it is possible that the reduced expression of SREBP-2 during ER stress contributes, at least partially, to the down-regulation of genes involved in thyroid hormone synthesis in FRTL-5 thyrocytes treated with TM.

## Conclusion

In conclusion, our findings show that the expression of key genes involved in thyroid hormone synthesis and their critical transcriptional regulators is attenuated in thyrocytes during mild ER stress, which is not associated with induction of apoptosis. Since the expression of TSHR was also reduced in thyrocytes under ER stress conditions and both, genes involved in thyroid hormone synthesis and transcriptional regulators of these genes are subject to regulation by TSH, we suggest that ER stress causes down-regulation of NIS, TG and TPO through impaired TSH/TSHR signaling in concert with reduced expression of TTF-1, TTF-2, PAX-8 and SREBP-2. Our observation that ER stress causes down-regulation of genes involved in thyroid hormone synthesis in thyrocytes might provide a mechanistic link between the presence of ER stress-induced UPR in human congenital hypothyroid goiter [11], in a mouse model of congenital hypothyroid goiter [12,13] and in cystinotic thyroids in a mouse model of cystinosis developing hypothyroidism [14].
